# 

**Published:** 1876-04

**Authors:** 


### CONTENTS:

### ORIGINAL COMMUNICATIONS.

ARTICLE I.—Address. By W. W. H. Thackston, M. D., D. D. S. .... 529

ARTICLE II.—"Dental Legislation," Review of an address by Dr. C. W. Barrett. By Prof. James B. Hodgkin, D. D. S. .... 538

ARTICLE III—Gold Screws for Retaining Fillings. By Dr. K. B. Davis .... 548

ARTICLE IV—The American Academy of Dental Surgery .... 552

ARTICLE V.—Alumni Meeting .... 558

ARTICLE VI.—Thirty-sixth Annual Commencement of the Baltimore College of Dental Surgery .... 561

### SELECTED ARTICLES.

ARTICLE VII.—Filling Teeth with Tin. By H. L. Ambler, M. D., D. D. S. .... 562

### EDITORIAL, ETC.

Exhaustive Effects of Dental Practice .... 566
Dental Education .... 567

### MONTHLY SUMMARY.

Radical Cure of Salivary Fistula .... 568
Morphia in Neuralgia .... 570
A New Solvent for Salicylic Acid .... 571
How to Prevent Chloroform Asphyxia .... 571
To Retard the Setting of Gypsum .... 572
Eczema Produced by Tincture of Arnica .... 572
A New Pair of Siamese Twins .... 573
Death from Ether .... 573
Solvents for Rubber .... 574
A Third Dentition at the Age of Seventy-three .... 575
How to Remove Stains from Steel Instruments .... 575
Toothache .... 575
Treatment of Diphtheria .... 575

